# Effect of Age, Breed, and Sex on the Health-Related Quality of Life of Owner Assessed Healthy Dogs

**DOI:** 10.3389/fvets.2021.603139

**Published:** 2021-02-05

**Authors:** Susan Rodger, E Marian Scott, Andrea Nolan, Andrea K Wright, Jacqueline Reid

**Affiliations:** ^1^School of Veterinary Medicine, University of Glasgow, Glasgow, United Kingdom; ^2^NewMetrica Research, Glasgow, United Kingdom; ^3^School of Mathematics and Statistics, University of Glasgow, Glasgow, United Kingdom; ^4^Edinburgh Napier University, Edinburgh, United Kingdom; ^5^Outcomes Research, International Centre of Excellence, Zoetis, Dublin, Ireland

**Keywords:** health-related quality of life, dogs, breed, sex, age, owner opinion, health status

## Abstract

Using an app, this exploratory study generated information on HRQL in a large cohort of dogs deemed healthy according to the owner. It forms the basis for further studies investigating the natural history of HRQL of dogs to inform the interpretation of interventional studies, but highlights the risks of relying on owner impression of health status. A previously published health-related quality of life (HRQL) instrument (VetMetrica™) that generates scores in four domains of quality of life in dogs - Energetic and Enthusiastic (E/E), Happy and Content (H/C), Active and Comfortable (A/C), and Calm and Relaxed (C/R), generated information on HRQL in 4,217 dogs (3 months−21 years). Dogs were categorized by age; young, 3–47 months, middle-aged, 48–95 months, and old, 96 months and older. Owners considered 2,959 dogs (3–95 months) to be “in perfect health” and these were used to explore the relationship between age, sex, breed and HRQL in apparently healthy dogs. Mean score was significantly greater (better) in young compared to middle-aged dogs in E/E, H/C and A/C and declined with advancing age. In H/C there was a small but significant difference in mean score between female and male dogs (mean greater in females), with a similar rate of decline in each gender with advancing age. In E/E there were very small but statistically significant differences in mean scores between certain breeds. In A/C there was a statistically significant interaction between breed and age and the rate of decline with advancing age differed with breed. Overall, age, breed, and sex predicted very little of the variation seen in HRQL scores. Data from a subset of 152 dogs, for whom clinical information was available, were used to examine the agreement between clinical evidence and owner opinion. According to the clinical records, 89 dogs were healthy and 63 had evidence of chronic disease. There was an approximately 40% disagreement between owner opinion on health status and clinical evidence of chronic disease (35% disagreement in all dogs and 43% in old dogs). HRQL scores were generally higher in dogs for whom there was no evidence of disease in the clinical record.

## Introduction

Quality of life (QOL) is a general term used in a variety of disciplines in which it is accepted that QOL is, like pain, a multi-dimensional construct that is subjectively experienced by and is uniquely personal to the individual. Health-related quality of life (HRQL) is the subjective evaluation of circumstances that include an altered health state and related interventions.

HRQL instruments can be disease-specific focusing on particular disorders or populations, or they can be generic, designed to be used in a variety of contexts and across a wide range of disease conditions. Generic instruments generate either a single index score or a health profile, which attempts to measure all important aspects of HRQL. Health profiles offer significant advantages – they allow the measurement of the effects of a disease state or its treatment on different dimensions of HRQL e.g., physical or emotional components and they can be applied to any population, sick or healthy. There are several profile measures available to measure HRQL in humans and one of the most popular, the Medical Outcomes Study-Short Form (SF-36) is a generic instrument that generates scores in eight domains of QOL (physical functioning, physical role limitations, bodily pain, general health perceptions, energy/vitality, social functioning, emotional role limitations, and mental health) which can be combined to produce two summary scores in physical and emotional health (1). The authors have previously reported the development of an owner reported 46 item generic profile measure to evaluate HRQL in dogs (VetMetrica™) (2), and subsequently this instrument was shortened to 22 items in order to facilitate its presentation via a smartphone app (3). This instrument generates scores in four domains of QOL - energetic/enthusiastic (E/E), happy/content (H/C), active/comfortable (A/C), and calm/relaxed (C/R) which, like the SF-36, can be combined to create summary scores in physical and emotional health.

The advantages of generic HRQL scales are many, including the comparisons of different disorders, disease severities and treatments. They also can measure the burden of chronic disease in populations as compared with healthy ones (4). However, despite its widespread use among people with chronic conditions, there have been few studies regarding the ways in which the SF-36 performs among healthy populations – see (5) for a useful review. Accordingly it has been difficult to estimate within-person changes that may be the consequence of natural aging. Such norms are important to establish because the effect of any intervention in a sick population may be confounded by changes due to the natural progression of HRQOL over time. Studies using the SF-36 have shown that the SF-36 is reliable and able to detect differences between groups defined by age and gender which are known to impact HRQL scale scores (6). A subsequent study showed that in healthy people emotional health improves with age while physical functioning scores decline, with women scoring consistently lower than men in each age group, with the exception of the general health perception domain (7). To the authors' knowledge there is no information available on HRQL in populations of healthy pet animals in relation to such biological variables, and one aim of this study was to generate such information to expand the body of knowledge in relation to the natural history of HRQL in companion animals.

In human health care, mobile health (m-health) applications are increasing and used in a diverse range of practices (8), but app technology has remained relatively under-exploited in veterinary medicine to date. There are numerous apps available to track HRQL in humans, including some specific to disease states such as cancer and mental health (9–12). The advent of app technology provides a unique opportunity for veterinary surgeons to obtain information about the animals under their care remotely, and although apps have been used in the management of epilepsy (13) and in parasite monitoring in dogs (14), the majority of health and lifestyle apps that are available offer only the ability to record and diarise activity or medical information. One exception is the PetDialog app (Zoetis Inc.) for companion animals which allows owners to record and monitor exercise, nutritional intake and socialization, but also gives them access, through their veterinary surgeon, to the VetMetrica™ HRQL tool for dogs (15). This enables the veterinary surgeon to gather data relevant to animal health and well-being from owners outwith the veterinary consultation, including HRQL data.

The objectives of this work were first to report the effects, if any, of age, sex, and breed on the HRQL of a large cohort of apparently healthy dogs, using app technology to obtain owner responses to a previously validated generic profile measure of HRQL (Study 1), and second to examine the concordance between clinical records, owner opinion and HRQL in dogs (Study 2).

## Materials and Methods

### Data Collection

Health-related quality of life (HRQL) data were collected from dog owners using a validated instrument (Vetmetrica™) (3) via a smartphone app (PetDialog, Zoetis). The HRQL instrument was incorporated as one of several features in the PetDialog app, which also required owners to input their dog's date of birth, breed, sex, and neutering status. Engagement with the HRQL instrument was entirely at the discretion of owners and uptake was not assessed. The PetDialog app was made available to pet owners in the United Kingdom and The Netherlands via 211 veterinary practices and was only accessible using a unique practice code. Due to data protection regulations, the data were anonymized such that the owners' demographic details and geographical location were unknown. These data were screened and where owners had completed multiple assessments for their dog, all except the most recent entry were removed. The Vetmetrica™ HRQL instrument contains 22 items, each of which comprises a descriptor (e.g., “active”) with a 7-point Likert rating scale, 0–6 (with 0 meaning “not at all” and 6 meaning “could not be more”), which are used to determine an HRQL score in each of the four domains E/E, H/C, A/C, and C/R (3). An additional question directed at assessing owner opinion was included alongside the 22 items (“Is your dog in perfect health – yes or no?”).

### Statistical Methods

Data were analyzed using Minitab® Statistical Software (2010) (Computer software). State College, PA: Minitab, Inc. (www.minitab.com). ANOVA, ANCOVA and General Linear Modeling (GLM) were used. The level of statistical significance was set at 5% (*p* < 0.05) for all analyses.

Study 1: To assess the effects of age (as a factor) and sex, for each HRQL domain, a GLM was fitted, adding age and sex as main effects and including interactions. Terms which were not statistically significant were removed and the model was re-fitted.

The Tukey HSD test was used as a *post-hoc* test to assess factor level differences. An identical procedure was followed using Age in months (as a continuous variable) using an analysis of covariance (ANCOVA). In order to explore the effect of breed, a subset of data from dogs belonging to those breeds with the greatest representation (*n* ≥ 30) were analyzed. For each HRQL domain, a linear model (ANOVA) was fitted, with Age segment, sex, and Breed and their second order interactions. Subsequently, terms which were not statistically significant were removed and the model was re-fitted.

Study 2: Chi-Squared Tests of Association. A Chi-squared test of association was used to assess the association between owner opinion regarding health and clinical record. Follow up confidence intervals for differences in means were used to look at the effect of veterinary assessed health status.

### Study 1: HRQL in a Healthy Cohort of Dogs

The aim of these analyses was to examine HRQL in a cohort of healthy dogs. Dogs were categorized as “healthy” or “unhealthy” according to owner opinion and those considered unhealthy (owners answered “No” to the question “Is your dog in perfect health?”) were excluded from this study. Due to the fact that the overall prevalence of non-infectious disease increases with age, it was assumed that elderly dogs are more likely to be unhealthy and therefore, in order to reduce the likelihood of including unhealthy dogs in these analyses, data from dogs aged 8 years or 96 months and above were also excluded. The effect of age was considered in two ways, firstly using “Age segment” as a factor whereby dogs aged 3–47 months of age were classified as “young” and dogs aged 48–95 months were classified as “middle-aged” and secondly, as a continuous variable (“Age in months”). The choice of boundaries for age categorization was based on the authors' professional veterinary opinion and clinical experience. It was not intended to define young, middle and old age in dogs but rather as an exploratory analysis of the dataset.

### Study 2. Agreement Between Clinical and Owner Opinions on a Subset of Dogs

For most dogs in the database, the basis of health assessment was owner opinion. However, in association with their veterinary practice clinical information was obtained for a small cohort of dogs. These data were accessed and assessed by an independent veterinary surgeon who visited each practice and searched the clinical records for entries made within 6 months of an owner HRQL assessment. All data were anonymised and apart from the owner's surname which was used to help identify individual dogs, no personal details of the owner were used. These dogs were obtained by random sampling from eight practices with the greatest compliance in data collection and met the following criteria: veterinary practice represented by at least 50 dogs in the dataset; the sample would have a ratio 1:2 of dogs in perfect health and not in perfect health state (owner's opinion); owner and veterinary assessments conducted within 6 months of each other. From these observations, relevant clinical information about chronic disease conditions diagnosed by the examining veterinary surgeon was extracted. Where there was no record of any chronic disease, the dog was classified as being “in perfect health,” whereas any evidence of disease in the notes was taken to mean that the dog was “not in perfect health.” This study had two objectives, first to assess the concordance between owner and clinical record, second to explore the differences if any between the clinically defined healthy and not healthy dogs.

## Results

### Study 1. HRQL in a Healthy Cohort of Dogs

**Figure 1:**
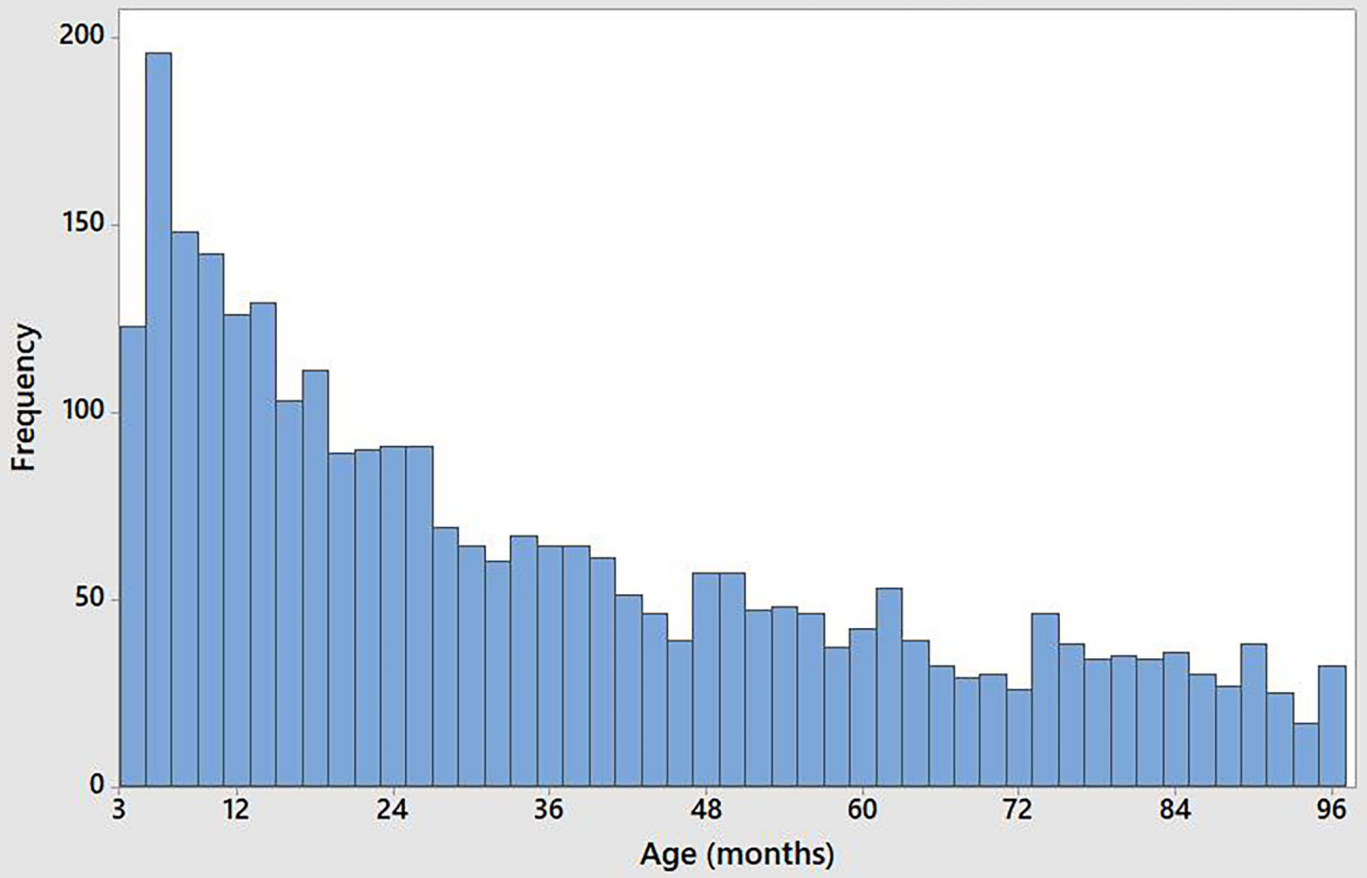
Age distribution of 2,959 dogs aged under 96 months (8 years) of age considered to be in perfect health by their owners.

Out of a total of 4,217 dogs in the full data set, 3,411 were in “perfect health” according to owner opinion. The dogs considered healthy by owners ranged from 3 to 206 months (17 years) of age and 2,959 were under 96 months (8 years) of age (the majority aged between 3 and 48 months of age) (Figure 1). Of the 2,959 considered healthy and aged under 96 months, 1,592 (53.8%) were male, 1,367 (46.2%) were female and of these, 150 (9.42% of) males and 145 (10.60% of) females were neutered.

There were 135 breeds represented by at least one individual dog. There were 20 breeds or specific cross-breeds (i.e., where the dam and sire breeds were known) represented by at least 30 individual dogs (Table 1).

**Table 1:** Dog breeds represented by at least 30 individuals.

**Breed**	**Male**	**Female**	**Total**
Retriever - Labrador	159	120	279
Spaniel - English Cocker	103	77	180
Jack/Parson Russell Terrier	77	59	136
Border Collie	65	59	124
Spaniel - English Springer	66	56	122
Cockapoo/Cockerpoo/Spoodle (X Spaniel/Poodle)	64	40	104
Bull Terrier - Staffordshire	51	44	95
German Shepherd/Alsatian	39	38	77
Retriever - Golden	45	32	77
Shih Tzu	40	31	71
Pug	48	19	67
Yorkshire Terrier	33	30	63
Border Terrier	27	28	55
Schnauzer (Miniature)	19	29	48
West Highland White Terrier	24	19	43
King Charles Spaniel - Cavalier	18	24	42
Lhasa Apso	17	21	38
Labradoodle (X Labrador/Poodle)	20	14	34
Beagle	21	12	33
Boxer	15	15	30
**Total**	**951**	**767**	**1718**

**Figure 2:**
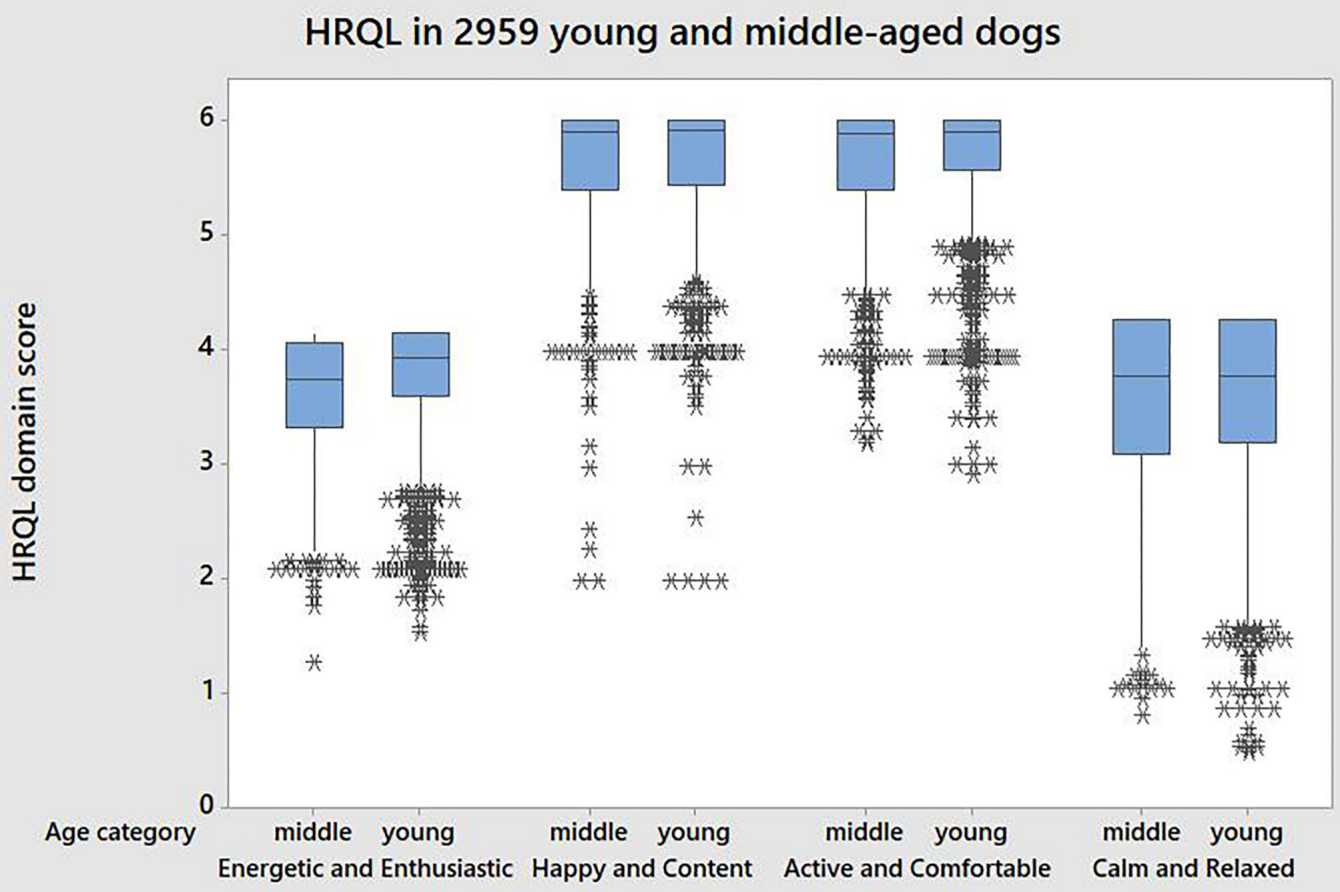
Profile of HRQL domain scores in 2,959 dogs considered in perfect health according to owners. Young refers to dogs aged 3–47 months; middle aged refers to dogs aged 48–95 months.

The profile of HRQL domain scores amongst young and middle-aged dogs in perfect health according to owner opinion is shown in Figure 2 with descriptive statistics shown in Table 2. These show the differences in mean domain score by age group, with the older group having lower mean scores on average (E/E *p* ≤ 0.001; H/C *p* = 0.002; A/C *p* ≤ 0.001). There is also clearly variability in the scores, with a large number of potential outliers (identified in Figure 2 by ^*^). For two of the domains (H/C, A/C) the maximum score of 6 is frequently recorded showing that there are ceiling effects in these domains.

**Table 2:** HRQL domain scores across 2959 young and middle-aged dogs considered in perfect health according to owner.

	**Mean**	SE mean^*^	Std dev^**^	Q1^***^	**median**	Q3^****^	IQR^*****^	***n***
**Energetic/enthusiastic**								
young	3.78	0.01	0.46	3.60	3.93	4.15	0.55	2,045
middle-aged	3.62	0.02	0.52	3.32	3.75	4.07	0.75	914
**Happy/content**								
young	5.69	0.01	0.48	5.44	5.92	6.00	0.56	2,054
middle-aged	5.63	0.02	0.53	5.39	5.90	6.00	0.61	914
**Active/comfortable**								
young	5.72	0.01	0.48	5.57	5.91	6.00	0.43	2,054
middle-aged	5.62	0.02	0.54	5.40	5.89	6.00	0.60	914
**Calm/relaxed**								
young	3.58	0.02	0.77	3.19	3.77	4.26	1.07	2,054
middle-aged	3.57	0.03	0.77	3.10	3.77	4.26	1.16	914

* *Standard error mean*,

** *standard deviation*,

*** *quartile 1*,

**** *quartile 3*,

***** *inter-quartile range*.

*Young refers to dogs aged 3–47 months; middle aged refers to dogs aged 48–95 months*.

A summary of the results of the statistical analysis using the linear models for each domain is as follows with detail provided in Table 3.

**Table 3:** Summary of significant effects of Age, Sex and Breed on HRQL domains.

	**Energetic and Enthusiastic**	**Happy and Content**	**Active and Comfortable**	**Calm and Relaxed**
**Age category**	Mean score Young > Mean score Middle-aged (*p* ≤ 0.001); Average difference between scores in Young and Middle-aged dogs = 0.16	Mean score Young > Mean score Middle-aged (*p* ≤ 0.002); Average difference between scores in Young and Middle-aged dogs = 0.06	Mean score Young > Mean score Middle-aged (*p* ≤ 0.001); Average difference between scores in Young and Middle-aged dogs = 0.10	No significant difference in mean score; Average difference between scores in Young and Middle-aged dogs = 0.01
**Age as continuous covariate**	Mean score declined with advancing age at a rate of 0.003582 per month (R-sq adjusted = 3.65%); predicted decline in score over 12 months = 0.04	Mean score declined with advancing age at a rate of 0.001515 per month (R-sq adjusted = 0.71%); predicted decline in score over 12 months = 0.02	Mean score declined with advancing age at a rate of 0.002217 per month (R-sq adjusted 1.3%); predicted decline in score over 12 months = 0.03	Not statistically significant
**Sex**	No significant difference in mean score	Mean score Female > Mean score Male (*p* = 0.039)	No significant difference in mean score	No significant difference in mean score
**Breed**	No significant difference in mean score	No significant difference in mean score	Significant difference in rate of decline of mean score with advancing age amongst different breeds; predicted decline in score over 12 months ranged from 0.01 to 0.12 in all breeds;	No significant difference in mean score

**Figure 3:**
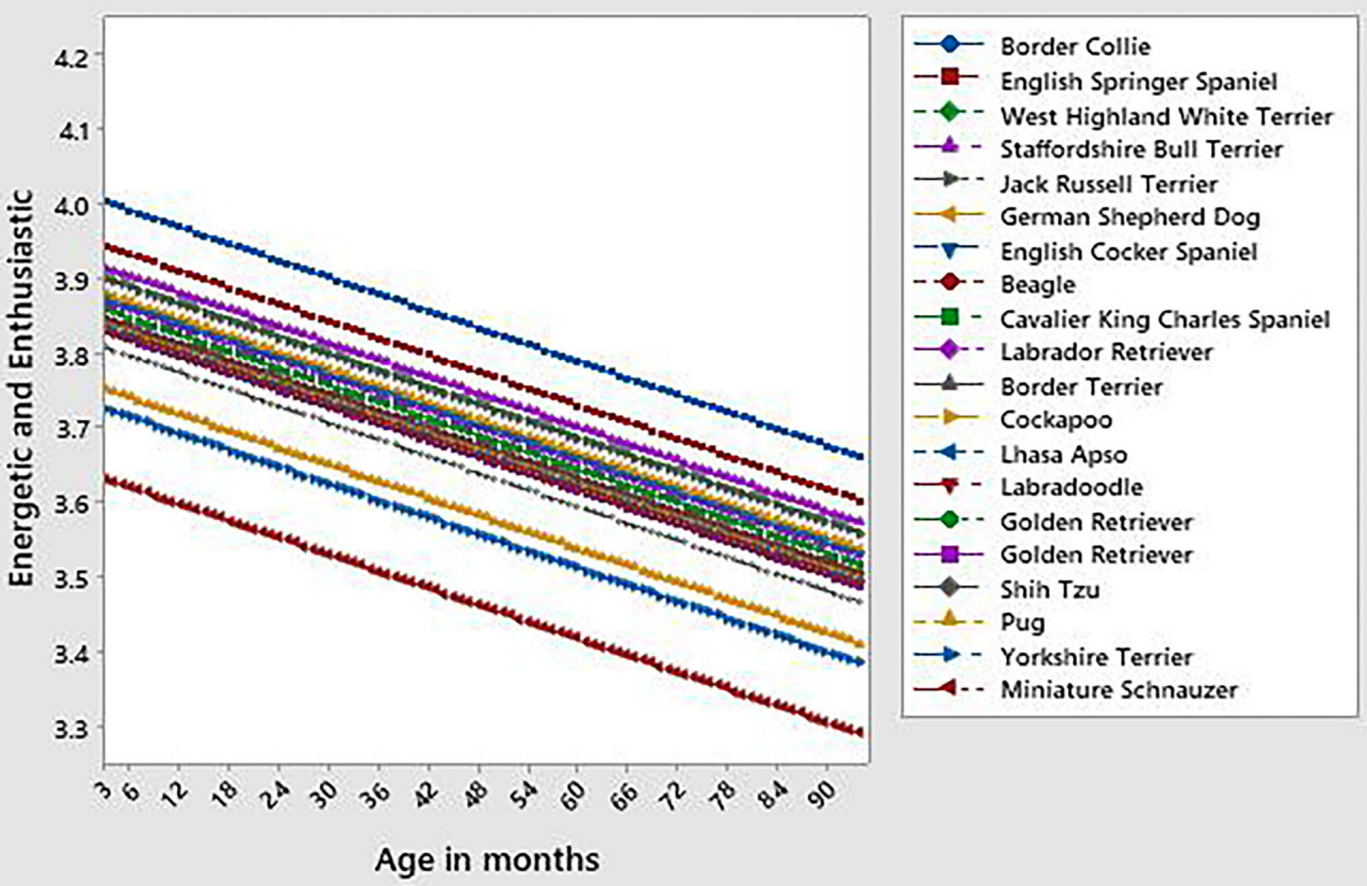
Regression model of the “Energetic and Enthusiastic” HRQL domain, according to age in months and breed for 20 breeds of dog, each represented by at least 30 individuals.

There were no statistically significant effects found for CR. For the three other HRQL domains, there was a statistically significant effect of age, both as a factor and as a continuous covariate, with older dogs having lower scores Breed was found to be statistically significant for E/E and A/C, but not for HC. Sex was only found to be statistically significant for HC. There was statistically significant evidence of declining HRQL scores with age, which for AC only included a breed effect (i.e., different breeds declined at different rates). For A/C the annual rate of decline in all breeds ranged from 0.01 to 0.12). Figure 3 shows the regression model for the domain E/E. It shows the breed differences on average, as well as the same rate of decline with advancing age. For a healthy border collie, over 10 years, its E/E score would be expected to drop from 4 to 3.6 on average. In all domains, the model variation explained was low (R-sq = 6.35%; 4.42% for E/E and A/C, respectively), leaving a large amount of the variation in HRQL domains unexplained.

### Study 2: Owner Opinion on Health Status vs. Clinical Data

Of the 152 dogs for whom clinical data were available (3.60% of total dogs), 49 (32.2%) were aged 96 months and older and 103 (67.8%) were aged between 3 and 95 months of age. For each of these 152 dogs, the owner responses and presence or absence of chronic disease in the clinical records are shown in Table 3. Overall, the level of agreement between owner opinion on “Is your dog/the dog in perfect health, yes or no?” and the presence or absence of disease according to clinical notes was 65.8% (100/152 dogs), whilst in old dogs it was 57.1% (28/49 dogs). The level of disagreement in older dogs compared to younger dogs was not statistically significant according to Pearson Chi-square test (Chi-square = 2.402, DF = 1, *p* = 0.121). In most cases where there was a disagreement, the owner answered “Yes” whilst the clinical notes indicated that disease was present (20.4% of all dogs and 28.6% of old dogs). However, there were also 21 dogs considered not to be in perfect health by owners for whom there was no record of disease in the veterinary clinical notes (13.8% of all dogs and 14.3% of old dogs).

### HRQL and Clinical Records

From the 152 dogs in the subsample with clinical records, there were 63 dogs with evidence of disease according to the clinical record and 89 without (Table 4). The diseases recorded included degenerative joint disease, obesity, cancer, skin disease, cardiac disease, neurological disease, ear disease, dental disease, respiratory disease, eye disease, gastro-intestinal disease, musculoskeletal disease and other medical conditions that did not fit in these categories. For some dogs, a severity was recorded e.g., mild chronic skin disease but for others, the severity was not evident. There were 30 dogs with evidence of degenerative joint disease, ranging in severity from mild to severe and many dogs had evidence of disease in multiple categories e.g., degenerative joint disease with concurrent obesity, skin disease and dental disease etc. Descriptive statistics for HRQL domain scores in dogs with clinical data are shown in Table 5. These data suggest that mean HRQL domain scores were generally higher in dogs for whom there was no evidence of disease on the clinical record. There was a statistically significant difference in mean score for “Active and Comfortable” (*p* ≤ 0.001). However, there was no statistically significant difference in mean score between these groups for Energetic and Enthusiastic (*p* = 0.255), Happy and Content (*p* = 0.163) or Calm and Relaxed (*p* = 0.433).

**Table 4:** Owner Opinion on their Dog's Health Status versus Clinical Evidence.

	**Evidence of disease in case record**		**No evidence of disease in case record**	
	**All ages**	**Old**	**All ages**	**Old**
Owner said dog is not in perfect health	32	18	**21**	**7**
	21.1%	36.7%	**13.8%**	**14.3%**
Owner said dog is in perfect health	**31**	**14**	68	10
	**20.4%**	**28.6%**	44.7%	20.4%
Total	63	32	89	17
	41.4%	65.3%	58.6%	34.7%

*Bold indicates where there was disagreement between owner opinion of health status and clinical evidence*.

**Table 5:** HRQL domain scores in 152 dogs for whom clinical data was available.

	**Mean**	SE mean^*^	Std dev^**^	**Median**	IQR^***^	***n***
**Energetic and enthusiastic**						
Disease recorded	3.10	0.10	0.76	3.18	1.06	63
No disease recorded	3.24	0.08	0.76	3.45	0.93	89
Difference in mean scores (95% Confidence Interval)	0.143 (−0.105, 0.391) *p* = 0.255					
**Happy and content**						
Disease recorded	5.15	0.10	0.79	5.30	0.96	63
No disease recorded	5.33	0.08	0.75	5.45	0.94	89
Difference in mean scores (95% Confidence Interval)	0.178 (−0.073, 0.430) *p* = 0.163					
**Active and comfortable**						
Disease recorded	4.59	0.11	0.90	4.60	1.45	63
No disease recorded	5.26	0.08	0.73	5.41	1.04	89
Difference in mean scores (95% Confidence Interval)	0.675 (0.404, 0.946) *p* ≤ 0.001					
**Calm and relaxed**						
Disease recorded	3.12	0.11	0.88	3.20	1.26	63
No disease recorded	3.23	0.10	0.93	3.43	1.55	89
Difference in mean scores (95% Confidence Interval)	0.117 (−0.177, 0.410) *p* = 0.433					

* *Standard error mean*,

** *standard deviation*,

*** *quartile 1*,

**** *quartile 3*,

***** *inter-quartile range*.

## Discussion

The objectives of this work were to report the effects, if any, of age, sex, and breed on the HRQL of a large cohort of apparently healthy dogs, using app technology to obtain owner responses to a previously validated generic profile measure of HRQL, and then to examine the concordance between clinical records, owner opinion and HRQL in dogs.

According to the data obtained via the PetDialog app, there is evidence of some variation in three of the HRQL domains (Energetic and Enthusiastic, Happy and Content and Active and Comfortable) according to the explanatory variables studied. In each of these domains, age was a significant factor - there was a general decline in score with advancing age, albeit that the rate of decline was very low. This is understandable given that the prevalence of health problems is higher in older animals and a corresponding decline in HRQL would therefore be expected. It is also in line with similar findings in human studies where older subjects tend to attain slightly lower mean scores for HRQL domains such as physical wellbeing, energy/vitality, social wellbeing and pain than younger adults (6). The fact that the fourth factor (Calm and Relaxed) does not show a similar trend is not unexpected because it shows more variability in healthy dogs than was apparent for the other three domains, which is perhaps not surprising given the spectrum of excitability in the healthy dog (3).

In companion animal studies, the effect of sex can be difficult to establish because of the high number of animals that are neutered. However, in this study 91% of males and 89% of females were entire, possibly as a consequence of the young age of many of the dogs. Sex was a statistically significant factor in one domain only (Happy and Content), with females scoring slightly higher on average than males. This is in contrast to studies in human healthcare where women have reported poorer health on all variables of the SF36 than did men (*p* < 0.001) except for general health perception (6). However, in the study reported here it should be noted that the magnitude of difference in mean score was very small and therefore probably of little practical significance.

There was some evidence of significant breed differences in HRQL score in the domains “Energetic and Enthusiastic” and “Active and Comfortable,” where small differences in mean score between breeds were found. The rate of decline with advancing age in “Energetic and Enthusiastic” was the same for each breed group. However, for “Active and Comfortable” the rate of decline in HRQL with advancing age was greater in certain breeds. This finding is, perhaps, to be expected, given that there are well documented breed differences in longevity (16, 17) and therefore those breeds of dog with shorter average lifespans would perhaps be expected to develop age-related health issues that affect activity at a younger age. Unfortunately there were no Great Danes, whose short lifespan is attributed to faster aging (18), represented. In general, these data suggest that there is a decline in score with advancing age in most breeds and some variability in the rate of decline between breeds. Despite the low breed numbers and restricted breeds studied, the authors consider that these data suggest that further investigation of the effect of breed on HRQL/aging is warranted. For example, in this limited study the mean (confidence intervals) annual rate of decline in healthy dogs for A/C across breeds was 0.03 (−0.05, −0.01) and a notably faster rate of decline could be indicative of an underlying asymptomatic health issue.

There remained considerable variability in HRQL domain scores unexplained by age, sex, and breed in this owner identified “perfect health” sample of dogs, for whom it is likely, given the results of study 2, that as many as 20% may have had underlying health conditions not recognized by the owner. It has been demonstrated that dog owners often underestimate the impact of health issues on the well-being of their pet, even where they are aware of their clinical signs and have knowledge of veterinary interventions. The complexity of the relationship between dog owners and their pets has been cited as one of the reasons for this apparent contradiction, with owners' emotions potentially influencing their response when asked to comment their dog's state of health (19, 20). Further, it has been suggested that owners' ability to recognize signs of ill-health in elderly dogs is particularly poor and that veterinary surgeons cannot rely on owners to report important signs of disease in these animals (21). The observation that owners often reported their dog to be “in perfect health” despite the presence of evidence to the contrary in the clinical records and further, that this applied more in owners of older dogs, is in concordance with these previous reports. It is interesting to note that in a study of childhood obesity, parents of overweight children invariably underestimated their children's weight, despite being knowledgeable regarding healthy eating patterns and fully conversant with the health risks associated with obesity (22).

In this study, chronic disease was chosen as the focus when clinical records were examined because of the likely associated slow rate of change in health status over time. However, the potential time difference of up to 6 months between owner assessment and clinical examination allows for the possibility that the dog's state of health was genuinely different at these assessments and may explain some of the variation between owner opinion and clinical evidence. It is also possible that despite suffering from a chronic disease, some dogs' clinical signs may have been well controlled such that owners perceived their health to be very good on the day of the assessment. Similarly, some dogs with no evidence of chronic disease according to the case records may have experienced minor acute trauma or self-limiting infectious disease close to the time of the owner assessment which did not necessitate a visit to the veterinary practice and was not therefore recorded. This may in part account for the large numbers of low score outliers shown in Figure 2. Nevertheless, it is possible that around 600 dogs in the 2,959 dogs classified as “in perfect health” by their owners may not have been, and this may have accounted for some of the unexplained variability in HRQL domain scores.

A question often asked of the authors, who developed VetMetrica™, is “Will the scoring be affected by the owner's mood?” and we have no data available to answer that question. Since the scale is developed with the express intention of decreasing respondent bias, it is to be hoped that any such effect is minimized (23). However, there is a growing literature to support the fact that dog behavior can be influenced by human emotion through facial, voice and olfactory cues (24–26) and therefore it is not inconceivable that on any one assessment day the owner's emotional state may have affected their dog's behavior sufficiently to affect the HRQL domain scores. Accordingly, the owner's emotional state could possibly be added to age, gender, breed, and health status as factors accounting for the variability in the HRQL domain scores in this study.

Although not statistically significant in 3 of 4 domains, the general trend toward greater mean HRQL domain score in those dogs with no clinical evidence of disease suggests that the instrument may be discriminatory. This concurs with previous findings where the tool was shown to distinguish healthy from sick dogs (2, 3). It is interesting to note that there was a statistically significant difference between dogs with evidence of disease and those without such evidence in the domain “Active and Comfortable.” This may be a reflection of the fact that 30 of the 63 dogs with evidence of disease were suffering from osteoarthritis which would be likely to affect their activity and pain levels.

The work described here was not a prospective study, but rather a retrospective analysis of data and the authors accept that there were clear limitations to their study. Dogs of 8 years and older were excluded from the analysis which is a significant limitation in the context of the investigation of natural aging. However, the fact that more than 1/4 of owners of these older dogs seemed to be incorrect in their interpretation of “perfect health” compared with 1/5 owners of the younger dogs suggests that the decision to restrict this study to dogs under 8 years was appropriate. In the younger cohort studied, the age distribution was heavily skewed to the right with the majority of the 2,959 dogs being between 3 months and 4 years of age. Furthermore, the owner impression of perfect health underpinned Study 1 and Study 2 demonstrated that this might be unreliable in ~20% of cases in the age group studied. A veterinary assessment of health status would have resulted in more dependable results, but this was not possible in this study. These limitations contribute to the fact that the results of Study 1 are not generalisable to the healthy dog population *per se*. Nevertheless, some of the findings in Study 1 regarding the effect of age, gender and breed on HRQL, though preliminary, suggest that some variation exists and that further study is warranted. Study 2 provides evidence to support the fact that owners cannot be relied upon to report accurately the health status of their dogs, especially when they are old. In cat studies the disagreement between owner and vet in terms of health status was 29 and 26% which is slightly higher than the 20% reported here for dogs (27). However, the cat study used direct vet clinical assessment compared with case record entries and this may account for the difference.

These limitations notwithstanding, to the authors' knowledge, this is the first study of its kind to generate any detailed information relative to HRQL in a large population of dogs, and additionally to collect data direct from owners by means of digital technology, to provide baseline HRQL data for future studies. The incorporation of the HRQL feature (VetMetrica™) in the PetDialog app for dogs was first reported in 2015 and since that time data has been gathered for over 9,000 dogs. The results described in this paper suggest that a large number of dog owners were willing to use an app to complete an HRQL questionnaire based on their dog's behavior. However, we have no evidence as to how many owners did not take the opportunity to complete the questionnaire on the app. Nevertheless, the authors are of the opinion that the study has shown the technology to be a useful means of owner engagement and a valid method of obtaining HRQL data remotely. This is significant because owner involvement is a key part of monitoring pet wellness and telehealth is likely to increase as veterinary practices increase their use of digital technologies post Covid19. By providing an app which is easy to use and accessible to owners it should be possible to obtain such information in future in order to track well-being in individual animals as well as for the purpose of surveillance, without reliance on owners and their pets requiring to attend the veterinary surgeon's premises.

In human healthcare it has been reported that in longitudinal studies within person declines (worsening health) with age were greater than estimated by cross sectional data alone (28). Accordingly, determining longitudinal within dog changes with age in a healthy cohort will be important in order to inform the interpretation of interventional studies conducted over time.

In conclusion, this exploratory study has generated valuable information regarding the natural history of HRQL in healthy dogs that lays the foundations for controlled studies to inform the interpretation of population studies as well as treatment effect in longitudinal interventional studies.

## Data Availability Statement

The original contributions presented in the study are included in the article/Supplementary Material, further inquiries can be directed to the corresponding author/s.

## Ethics Statement

Ethical review and approval was not required for the animal study because the Zoetis Ethical Review Panel deemed ethical permission was not needed on the basis that the ethical review process would not routinely include survey information that clients have provided about their pets. Written informed consent for participation was not obtained from the owners because Owners completed their HRQL assessments (survey relating to their dog's behavior) on a web platform after consenting to their data being used for research purposes.

## Author Contributions

ES, AN, and JR: conceptualization. AW: data provision. SR: writing. SR and ES: statistical analysis. All authors: review and editing.

## Conflict of Interest

JR was employed by the company NewMetrica Ltd and AW by Zoetis Inc. The remaining authors declare that the research was conducted in the absence of any commercial or financial relationships that could be construed as a potential conflict of interest.
